# 

**Published:** 1845-12

**Authors:** 


### NOTICE TO READERS AND CORRESPONDENTS.

We have to acknowledge, in addition to our usual exchanges, the receipt of the last number of the Transactions of the College of Physicians of Philadelphia. Notice of its contents we are obliged to defer to the next issue of the Journal.

A communication has been received from Dr. U. P. Golliday, which arrived too late for insertion. Ed.

